# Epidemiology and Molecular Typing of Pregnancy-Associated Listeriosis Cases in Lombardy, Italy, over a 10-Year Period (2005–2014)

**DOI:** 10.1155/2017/6479121

**Published:** 2017-03-19

**Authors:** Virginia Filipello, Ettore Amato, Maria Gori, Pol Huedo, Giulia Ciceri, Sara Lomonaco, Mirella Pontello

**Affiliations:** ^1^Department of Veterinary Sciences, Università degli Studi di Torino, Largo Braccini 2, 10095 Grugliasco, Italy; ^2^Department of Health Sciences, Università degli Studi di Milano, Via di Rudinì, 8 20142 Milano, Italy

## Abstract

In developed countries, pregnancy-related listeriosis accounts for 20–43% of total invasive listeriosis. This work describes the first pregnancy-related listeriosis survey in Italy based on two data sources, that is, mandatory notification system and regional laboratory-based network. Out of 610 listeriosis cases reported over a 10-year period, 40 were pregnancy-related (6.6%). Among these, 29 pregnancy-related isolates were available and have been analysed with serotyping, Pulsed-Field Gel Electrophoresis, and Multi-Virulence-Locus Sequence Typing. No maternal fatality was recorded, but 11 (29.7%) pregnancies resulted in a foetal death, a miscarriage, or a birth of a foetus dying immediately after birth. The average incidence of pregnancy-related listeriosis was 4.3 cases per 100000 births, and the proportion of pregnancy-associated listeriosis among ethnic minorities was significantly higher compared to the general population (30.0% versus 3.5%, *P* < 0.01).* L. monocytogenes* isolates belonged to serotypes 1/2a, 1/2b, and 4b, with the latter significantly more prevalent among pregnancy-related isolates. Twenty different pulsotypes were distinguished and 16 out of the 29 isolates were classified into seven clusters. A total of 16 virulence types (VTs) were identified. Five VTs accounted for 45% of the total cases and coincided with those of previously described Epidemic Clones (ECs) of* L. monocytogenes*.

## 1. Introduction

*Listeria monocytogenes *is a causal agent of invasive infections mostly of foodborne origin. Ready-to-eat foods, in particular, are unanimously considered high-risk sources of* L. monocytogenes *[1–4]. WHO estimated that worldwide in 2010 listeriosis resulted in 23150 illnesses, 5463 deaths, and 172823 disability-adjusted life-years (DALYs) [5]. In the European Union (EU) 1676 confirmed cases were reported in 2012, with an overall case rate of 0.39 per 100000 population and a predominance of cases among subjects over 65 years [6]. Notification of invasive listeriosis has been mandatory in Italy since 1990 and in 2012 the national rate (0.15 per 100000 population) was lower than most countries in the EU [6]. In the same year, however, the northern Italian region of Lombardy with approximately 10 million inhabitants showed a higher annual notification rate of 0.46 cases per 100000 inhabitants [7].

Invasive listeriosis mainly affects immunocompromised subjects, the elderly, pregnant women, and newborns [8]. Pregnant women are a critical target subpopulation for* L. monocytogenes* infection. Indeed, pregnancy has been associated with an 18-fold increased risk of developing disease than the nonpregnant female population [3, 9, 10]. Pregnancy-associated cases have also been more commonly detected among ethnic minorities [11]. Pregnant women may experience fever, miscarriage, premature delivery, or stillbirth but are frequently asymptomatic [3]. Consequently, it is likely that pregnancy-associated cases of listeriosis could go underdiagnosed, especially when infection occurs during the first trimester of pregnancy or the infection ends in an early abortion. Although women with comorbidities are at higher risk, most cases occur in otherwise healthy pregnant women [9, 12].

In recent years, epidemiology of pregnancy-related listeriosis has been investigated in several developed countries, such as England and Wales [13], France [14], United States [3], Netherlands [15], and Israel [16]. A proportion between 20.7% [5] and 43% [17] of* L. monocytogenes *invasive infections are related to pregnancy. A previous study on invasive listeriosis in Lombardy reported that about 11.0% of the confirmed cases were pregnancy-related [18]. The main indicator used to describe the annual incidence of pregnancy-related listeriosis is the rate of number of cases per number of live births. Between 1984 and 2011 this indicator in France fell from 60 to 5 cases per 100000 live births, which is a decline by a factor of 12 [14]. Conversely, in the years 1998–2007 the yearly incidence of pregnancy-associated listeriosis in Israel ranged from 5.5 to 25.2 cases per 100000 births [16]. In USA, application of preventive measures (“zero tolerance”) since 1985 resulted in a 44% reduction of perinatal listeriosis, from 17.4/100000 to 8.6/100000 live births [8].

Molecular subtyping has widely contributed to knowledge about epidemiology of* L. monocytogenes*, by characterizing isolates and tracking sources and transmission pathways, and has also helped in the establishment of more efficient prevention strategies. The gold-standard technique has traditionally been Pulsed-Field Gel Electrophoresis (PFGE) [19]. However, in recent years some complementary subtyping methods, such as Multilocus Sequence Typing (MLST) and Multi-Virulence-Locus Sequence Typing (MVLST), are being applied for epidemiological purposes [20–22]. In particular, MVLST allows the identification of Epidemic Clones (ECs), which are genetically related and presumably of a common ancestor isolates, which have been implicated in geographically and temporally unrelated outbreaks [23–25]. Only a few studies have approached the molecular characterization of* L. monocytogenes* isolates from pregnancy-related infection cases. The results of those studies suggest that pregnancy-related cases are not correlated with specific molecular types [16].

The present study was undertaken to describe the clinical and epidemiological characteristics of the pregnancy-associated cases recorded between 2005 and 2014 in Lombardy and the molecular features and virulence genes patterns of* L. monocytogenes* isolates.

## 2. Methods

### 2.1. Data Sources and Case Definition

Pregnancy-associated listeriosis cases from 2005 to 2014 were extracted from (i) the regional mandatory notification web-based infectious disease system (MAINF) [7] and (ii) the regional network for laboratory-based surveillance of* L. monocytogenes* infections (LabSS). MAINF collects all available information regarding pregnancy and nonpregnancy listeriosis cases (i.e., age, clinical symptoms, underlying disease, and outcome) in a database. LabSS is based on voluntary referral of clinical isolates to the Regional Reference Laboratory at the Department of Health Sciences, University of Milan.* L. monocytogenes* isolates from hospitalized clinical cases are collected and a standardized report form is used to collect demographic, clinical, and microbiological data.

Pregnancy-related listeriosis cases were defined according to notification criteria, namely, the presence of compatible clinical symptoms and isolation of* L. monocytogenes* from a clinical sample of pregnant woman or foetus, stillborn, and newborn aged < 28 days (MAINF). Additionally, a case was also defined based on the isolation of* L. monocytogenes* from a clinical sample of pregnant woman or foetus, stillborn, and newborn aged < 28 days (LabSS).

Confirmed cases with isolation of* L. monocytogenes* from both mother and infant were counted as single cases.

### 2.2. Characterization of L. monocytogenes Isolates

*L. monocytogenes* from listeriosis cases was isolated on selective media and confirmed by biochemical tests kit (API Listeria, bioMérieux) by the hospital laboratories participating in the surveillance network and subsequently referred to the Regional Reference Laboratory.

#### 2.2.1. Serotyping

Serotyping was performed on both pregnancy-related and non-pregnancy-related isolates at the Istituto Superiore di Sanità (Rome, Italy). A multiplex-PCR (MUX-PCR) assay was performed to separate* L. monocytogenes* strains into four different serogroups: 1/2a and 3a; 1/2c and 3c; 1/2b and 3b; and 4b, 4d, and 4e [27]. Subsequently, conventional serotyping was then used to confirm the specific serotype within the serogroups, using* Listeria* antisera (Denka Seiken Co., Ltd., Tokyo, Japan), according to manufacturer's instructions. For the purpose of the study isolates serotyped as serotype 4b/4e by seroagglutination were assumed to be as serotype 4b as the latter is the disease causing serotype.

#### 2.2.2. Pulsed-Field Gel Electrophoresis (PFGE)

PFGE was carried out on both pregnancy-related and non-pregnancy-related isolates according to the PulseNet protocol [19]. Briefly, DNA samples were first restricted using* Asc*I enzyme and then separated in a 1% agarose gel electrophoresis, using a PFGE CHEF-MAPPER® System (Bio-Rad).* Apa*I enzyme was used where the* Asc*I profiles were indistinguishable. Clustering analysis was performed with BioNumerics 6.6 software (Applied Maths, Sint-Martens-Latem, Belgium) by using the unweighted pair group-matching algorithm (UPGMA) and the Dice correlation coefficient with a tolerance of 1.5%. In this study, isolates were considered to belong to the same cluster when they had a similarity value ≥ 90%. Clusters were identified with a consecutive number as in the previous study [18].

#### 2.2.3. Multi-Virulence Locus Sequence Typing (MVLST)

MVSLT was carried out on all pregnancy-related isolates as previously described [23]. Intragenic regions of six virulence genes (*clpP*,* dal*,* inlB*,* inlC*,* lisR,* and* prfA*) were amplified and sequenced by IGA Technology Services (Udine, Italy), using ABI 3730XL DNA analyser. Multiple sequence alignments were performed using the Molecular Evolutionary Genetic Analysis software MEGA 6 [26] and then compared with sequences of previously typed* L. monocytogenes* available from the MVLST database [28]. New allelic sequences, that is, with at least one nucleotide difference, were attributed with arbitrary virulence types (VTs). A neighbor-joining tree was constructed using the neighbor-joining method in MEGA 6 [26] based upon the number of nucleotide differences.

### 2.3. Statistical Analysis

Either the *χ*-square test or the Fisher exact test (Epi-Info 6.04 software, Centers for Disease Control and Prevention, Atlanta, GA, US) was used to assess associations between categorical variables. Differences were considered statistically significant when *P* was <0.05.

## 3. Results and Discussion

### 3.1. Clinical and Epidemiological Data

Over the ten-year period under study (2005–2014), 610 cases of invasive listeriosis were recorded in Lombardy by integrating data from the two surveillance regional systems (MAINF and LabSS). Forty out of them (6.6%) were pregnancy-related. The median age of mothers was 35 years (range 17–45). The survival outcome was known in 37 cases (Table 1); among these, no maternal fatality was recorded, while 11 pregnancies (29.7%) resulted in foetal death, miscarriage or stillbirth. In 32 cases (80%), symptoms developed in women after the twenty-fifth week of pregnancy. Regarding foetal and neonatal cases, 21 cases occurred after the twenty-fourth week of pregnancy. All five cases occurring between the 13th and 24th week were fatal. In one fatal case the week of pregnancy was unknown.

**Figure 1:**
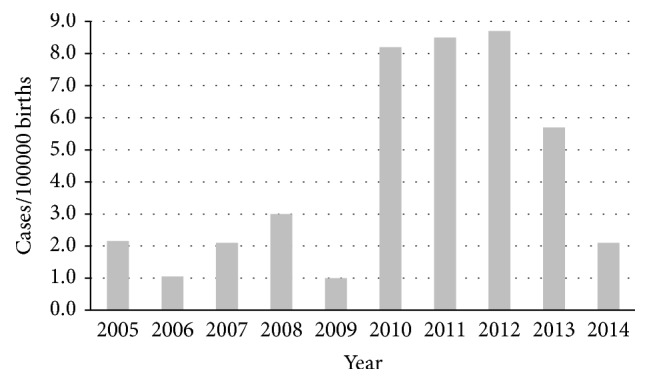
Number of pregnancy-related listeriosis per 100000 births observed in Lombardy, Italy, from 2005 to 2014.

**Figure 2:**
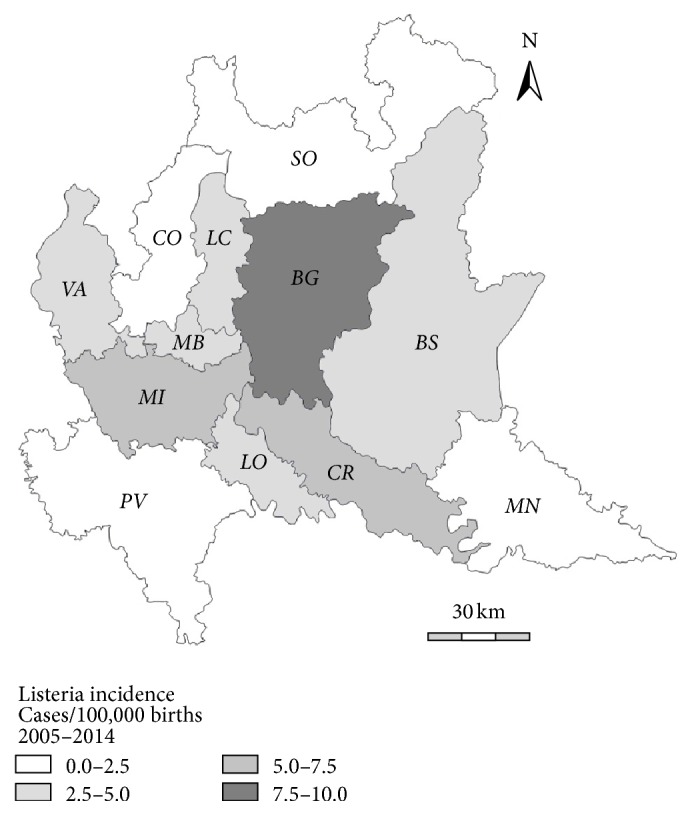
Geographical distribution of pregnancy-related listeriosis cases (per 100000 births) observed in Lombardy, Italy, from 2005 to 2014.* BG: Bergamo; BS: Brescia; CO: Como; CR: Cremona; LC: Lecco; LO: Lodi; MN: Mantua; MI: Milan; MB: Monza Brianza; PV: Pavia; SO: Sondrio; VA: Varese*.

The average incidence of pregnancy-related listeriosis in the ten-year period was 4.3 cases per 100000 births (ranging from 1.0 case per 100000 births in 2009 to 8.7 cases per 100000 births in 2012), as estimated based on population data from the Italian National Institute of Statistics (ISTAT) [29]. In the years 2010, 2011, and 2012, in particular, the incidence was almost doubled compared to the average incidence for the whole period (>8/100000) (Figure 1). Cases were observed in eight out of the 12 provinces of the Lombardy region, with the highest observed incidence in Bergamo followed by Cremona and Milan (9.7, 6.9, and 5.7 per 100000 births, resp.). In contrast, no listeriosis cases were recorded in the provinces of Como, Mantua, Pavia, and Sondrio (Figure 2). The proportion of pregnancy-related listeriosis among ethnic minorities (including immigrants from Ecuador, Peru, Albania, Hungary, Romania, Ukraine, Morocco and Saudi Arabia), was about ten times as higher as that of the general population (30.0% versus 3.5%) (*P* < 0.01).

### 3.2. Microbiological and Molecular Typing

**Figure 3:**
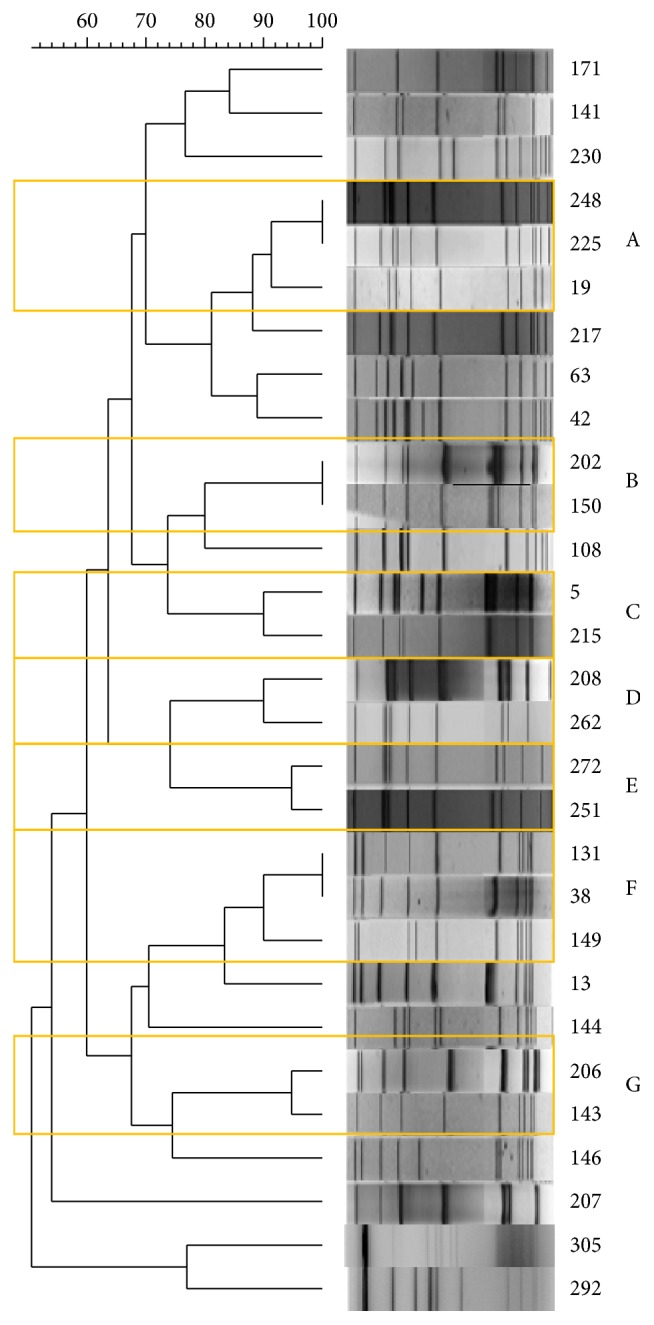
*Asc*I-PFGE restriction patterns of the 29 pregnancy-related isolates of* L. monocytogenes*, Lombardy, Italy, 2004–2015. Clusters with ≥90% similarity are highlighted by orange boxes and identified with a letter.

The results from microbiological and molecular typing are shown in Table 2. A total of 304* L. monocytogenes *isolates were collected; among these, 29 were pregnancy-related. The latter were serotyped as 1/2a (*n* = 15, 51.7%), 4b (*n* = 13, 44.8%), and 1/2b (*n* = 1, 3.5%). The 4b serotype was significantly more prevalent among pregnancy-related isolates (44.8% in pregnancy-related isolates versus 18.1% (*n* = 55) nonpregnancy isolates, *P* = 0.01). Twenty different* Asc*I pulsotypes were recognized.* Apa*I profiles of* Asc*I indistinguishable profiles did not confirm 100% similarity but highlighted a close correlation with a similarity percentage higher than 70%. Sixteen out of the 29 isolates were classified into seven clusters (Figure 3). When considering both pregnancy-related and non-pregnancy-related isolates, only cluster 6 showed a clear prevalence of pregnancy-related isolates (4/5; Table 2).

A total of 16 VTs were identified, 10 were unique, and 6 had more than one isolate, identified among the 29* L. monocytogenes* isolates. MVLST confirmed the relationship between the isolates belonging to clusters 3, 5, and 13, while the other clusters included isolates associated with different VTs. Overall, 48% (*n* = 12) of* L. monocytogenes* isolates showed VTs that had been previously associated with ECs.

**Figure 4:**
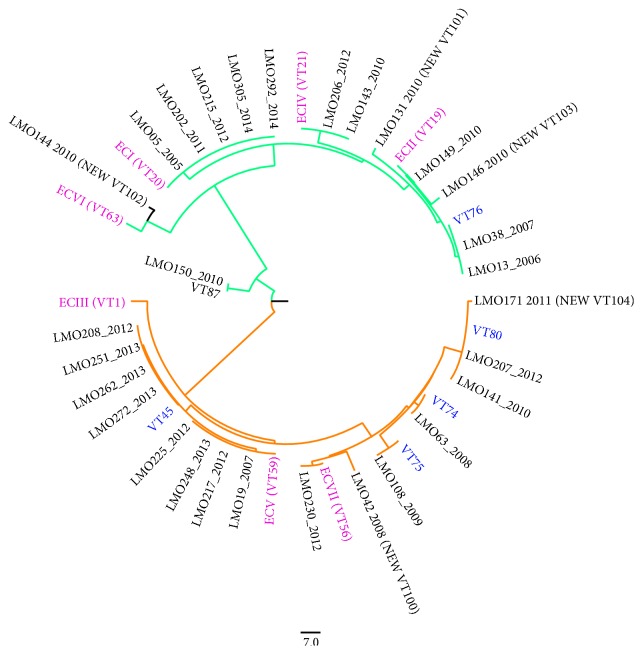
Unrooted neighbor-joining tree computed in MEGA6 [26] for Multi-Virulence-Locus Sequence Typing data obtained for the 29 pregnancy-related isolates of* Listeria monocytogenes* isolates collected in Lombardy, Italy, from 2005 to 2014. Green indicates isolates of serotype 4b and orange corresponds to isolates of serotype 1/2a. Epidemic Clones (ECs) and non-ECs virulence type (VT) reference strains are highlighted in pink and blue, respectively.

The neighbor-joining tree revealed two main groups that clearly separated according to serotype (Figure 4). The obtained gene sequences were deposited in the GenBank database [30] with the accession numbers: KP789770–KP789944.

A national Italian estimate of the proportion of pregnancy-related listeriosis cases at the national scale is unavailable. This study showed that 6.6% of the identified listeriosis cases in Lombardy were pregnancy-related and such frequency is lower compared with the rates in other countries (UK 12%; USA 16.9%; France 18%; Spain 32.5%; Israel 35%) [3, 11, 14, 16, 31] and the meta-analysis performed by Maertens de Noordhout et al. [5] (20.7%). It is not immediately clear whether this lower proportion is real or it is possibly due to underdiagnosis or underreporting issues. Underdiagnosis could be more likely, considering that most cases are diagnosed during the third semester, while earlier stages miscarriage may be seldom investigated. In fact, in this study no cases were reported in the first trimester and only five cases were reported during the second one. However, the percentage of pregnancy-related cases over the 10-year period increased to 10% of all listeriosis cases when only data from LabSS were considered. This value is similar to what was observed by Mammina et al., 2013 [18], and suggests a higher sensitivity of LabSS compared to MAINF in detecting pregnancy-related cases.

The age of the mother did not appear to be a risk factor for infection by* L. monocytogenes*. On the other hand, the correlation of infection with the presence of underlying conditions could not be precisely evaluated given the limited number of cases and the unavailability of some clinical information.

It is noteworthy that the proportion of pregnancy-related listeriosis among ethnic minorities was higher than the general population. Ethnicity-related variations in the risk of acquiring listeriosis are well documented and related to differences in diet or food consumption habits [32]. Moreover, incidence of pregnancy-associated listeriosis has been correlated with socioeconomic features (e.g., neighborhood deprivation) and food consumption behaviours (e.g., purchasing foods from convenience or local stores) both more frequent among ethnic minorities [11, 13].

In this study serotype 4b was significantly more prevalent among pregnancy-related cases compared to those non-pregnancy-related cases. This did not apparently impact the pregnancy outcome. Similar findings have been reported in other countries (Austria, 1997–2000; Israel, 1998–2007), with serotype 4b and specific PFGE types being associated with pregnancy-related listeriosis without any correlation between lineage and pregnancy outcome [16, 33]. In 1992 a large listeriosis outbreak in France was reported, which was attributed to serotype 4b and characterized by a high number of pregnancy-related cases (33%, 92 out of 279 total cases) [14].

Twenty-two out of the 29 isolates belong to eight clusters previously observed in a larger study on* L. monocytogenes* isolates from human clinical cases in Lombardy [18] (Table 2). The finding of clusters including both isolates from pregnancy-related cases and isolates from listeriosis cases not associated with pregnancy prompted the local health authorities to carry out an epidemiological investigation in order to possibly identify a food source for the pregnancy-related cases. No correlation with putative food sources was detected.

Clustering by PFGE and MVLST yielded a good concordance, with some exceptions. Using MVLST, different VTs were assigned to all the isolates grouped in cluster 2 and cluster 6. Interestingly, 13 isolates were assigned to five different ECs [23, 25]. A “predilection” of* L. monocytogenes* ECIV for pregnancy has been observed by a previous study [16], but this study does not seem to confirm that finding. In fact, 16 out of 29 isolates did not belong to any of the currently recognized ECs.

To guarantee a continuum with the previous study [18] the same parameters for the comparison analysis were applied. This led to discrepancies between profiles visibly not 100% similar and the software analysis (e.g., isolates 248 and 225; Figure 3) and is a limitation to the study. The interpretation of PFGE results is not always straightforward; therefore, sequence-based methods such as MVLST are advantageous because interpreting the results and sharing the data between laboratories are more reliable and accurate compared to band-based methods [23].

## 4. Conclusions

Although the study has been conducted only in Lombardy, this region of 10 million inhabitants accounts for 17% of the whole Italian population. However, because of major differences in migration patterns and food habits compared with other geographical areas, the results of this study can be generalized only for Northern Italy. Moreover, the regional surveillance systems are not harmonized and their organization varies widely between different regions and, in many cases, do not provide information about molecular subtyping of isolates.

Overall, the results of this study confirm that a “type” of* L. monocytogenes* specifically associated with pregnancy-related infections is not identifiable. In conclusion, this study highlights the public health importance of pregnancy-related listeriosis and the complexity of its epidemiological behaviour, as shown by the molecular analysis of isolates. The implementation of prevention strategies targeted at the ethnic minorities, with a special attention to food habits of pregnant women, is strongly advised.

## Figures and Tables

**Table 1:** Demographic and clinical data of the 40 pregnancy-related listeriosis observed in Lombardy, Italy from 2005 to 2014.

Characteristics	Number of cases (%)
Ethnicity	
Italian	28 (70.0)
Foreign	12 (30.0)
Gestational week	
1–12	0 (0)
13–24	5 (12.5)
>24	32 (80.0)
Unknown	3 (7.5)
Evidence of infection^*∗*^	
Only in the mother	17 (42.5)
Only in the foetus/newborn	19 (47.5)
Both	4 (10.0)
Outcome of pregnancy^±^	
Live birth	26 (70.3)
Stillbirth	4 (10.8)
Miscarriage	7 (18.9)
Fatality rate^±^	11 (29.7)

^*∗*^Sites of infection with a positive culture were as follows:

(i) Blood (7), placenta (5), liquor (1), others (7), and unknown sites (1) for maternal infection.

(ii) Blood (12), foetal exudate (1), faeces (1), gastric aspirates (1), ear (2), pharynx (2), skin (1), cerebrospinal fluid (1), rectum (1), and others (1) for foetal-neonatal listeriosis.

^±^The pregnancy outcome was known for 37 cases only.

**Table 2:** Serotyping and molecular subtyping results for the 29 pregnancy-related isolates under study (2005–2014, Lombardy, Italy).

Isolate	Year	Source	Serotype	PFGE			VT	EC
				Cluster	Cluster B^*∗*^	p/tc°		
LMO 150	2010	Foetus, intestinal content	4b	B	2	4/27	87	
LMO 202	2011	Mother, blood	4b				20	I
LMO 5	2005	Mother, blood	4b	C			20	I
LMO 215	2012	Foetus, exudate	4b				20	I

LMO 305	2014	Foetus, blood	4b	—	3	2/9	20	I
LMO 292	2014	Foetus, blood	4b	—			20	I

LMO 143	2010	Mother, blood	4b	G	5	2/15	21	IV
LMO 206	2011	Foetus, blood	4b				21	IV

LMO 13	2006	Foetus, pharynx	4b	—	6	4/5	76	
LMO 38	2007	Foetus, CSF^*Ø*^	4b	F			76	
LMO 131	2010	Mother, blood	4b				101	
LMO 149	2010	Foetus, pharynx	4b				19	II

LMO 63	2008	Foetus, pharynx	1/2a	—	8	1/12	74	

LMO 42	2008	Placenta	1/2a	—	9	5/30	100	
LMO 217	2012	Placenta	1/2a	—			59	V
LMO 19	2007	Foetus, gastric aspirate	1/2a	A			59	V
LMO 225	2012	Foetus, blood	1/2a				59	V
LMO 248	2013	Mother, blood	1/2a				59	V

LMO 108	2009	Foetus, blood	1/2a	—	U^⊗^		75	

LMO 230	2012	Placenta	1/2a	—	10	1/10	56	VII

LMO 141	2010	Placenta	1/2a	—	11	3/47	80	
LMO 171	2011	Foetus, blood	1/2a	—			104	
LMO 207	2012	Foetus, blood	1/2a	—			80	

LMO 208	2011	Foetus, blood	1/2a	D	New profile	4/18	45	
LMO 262	2013	Placenta	1/2a				45	
LMO 251	2013	Foetus, skin	1/2a	E			45	
LMO 272	2013	Foetus, skin	1/2a				45	

LMO 144	2010	Foetus, blood	1/2b	—	U^⊗^		102	

LMO 146	2010	Mother, blood	4b	—	U^⊗^		103	

VT: virulence type; EC: Epidemic Clone.

^*∗*^PFGE clusters obtained when considering all listeriosis cases between 2005 and 2014 (pregnancy-related and non-pregnancy-related) numbered as in Mammina et al., 2013 [18].

°p/tc: number of pregnancy-related cases/total cases.

^*Ø*^CSF: cerebrospinal fluid.

^⊗^U: Unclustered.

## References

[1] Cartwright E. J., Jackson K. A., Johnson S. D., Graves L. M., Silk B. J., Mahon B. E. (2013). Listeriosis outbreaks and associated food vehicles, United States, 1998–2008. *Emerging Infectious Diseases*.

[2] Lianou A., Sofos J. N. (2007). A review of the incidence and transmission of *Listeria monocytogenes* in ready-to-eat products in retail and food service environments. *Journal of Food Protection*.

[3] Jackson K. A., Iwamoto M., Swerdlow D. (2010). Pregnancy-associated listeriosis. *Epidemiology and Infection*.

[4] Mateus T., Silva J., Maia R. L., Teixeira P. (2013). Listeriosis during pregnancy: a public health concern. *ISRN Obstetrics and Gynecology*.

[5] Maertens de Noordhout C., Devleesschauwer B., Angulo F. J. (2014). The global burden of listeriosis: a systematic review and meta-analysis. *The Lancet Infectious Diseases*.

[6] European Centre for Disease Prevention and Control (2014). *Annual Epidemiological Report 2014—Food-and Waterborne Diseases and Zoonoses*.

[7] MAINF Surveillance, notification, control of infective diseases: revision and reorganization of preventive interventions in Lombardy Region.

[8] Allerberger F., Wagner M. (2010). Listeriosis: a resurgent foodborne infection. *Clinical Microbiology and Infection*.

[9] Lamont R. F., Sobel J., Mazaki-Tovi S. (2011). Listeriosis in human pregnancy: a systematic review. *Journal of Perinatal Medicine*.

[10] Awofisayo A., Amar C., Ruggles R. (2015). Pregnancy-associated listeriosis in England and Wales. *Epidemiology and Infection*.

[11] Mook P., Grant K. A., Little C. L., Kafatos G., Gillespie I. A. (2010). Emergence of pregnancy-related listeriosis amongst ethnic minorities in England and Wales. *Euro Surveillance*.

[12] Siegman-Igra Y., Levin R., Weinberger M. (2002). *Listeria monocytogenes* infection in Israel and review of cases worldwide. *Emerging Infectious Diseases*.

[13] Gillespie I. A., Mook P., Little C. L., Grant K. A., McLauchlin J. (2010). Human listeriosis in England, 2001–2007: association with neighbourhood deprivation. *Euro Surveillance*.

[14] Girard D., Leclercq A., Laurent E., Lecuit M., de Valk H., Goulet V. (2014). Pregnancy-related listeriosis in France, 1984 to 2011, with a focus on 606 cases from 1999 to 2011. *Euro Surveillance*.

[15] Doorduyn Y., De Jager C. M., Van Der Zwaluw W. K. (2006). Invasive Listeria monocytogenes infections in the Netherlands, 1995–2003. *European Journal of Clinical Microbiology & Infectious Diseases*.

[16] Elinav H., Hershko-Klement A., Valinsky L. (2014). Pregnancy-associated listeriosis: clinical characteristics and geospatial analysis of a 10-year period in Israel. *Clinical Infectious Diseases*.

[17] Food and Agriculture Organization of the United Nations/World Health Organization (2009). *Risk Characterization of Microbiological Hazards in Food*.

[18] Mammina C., Parisi A., Guaita A. (2013). Enhanced surveillance of invasive listeriosis in the Lombardy region, Italy, in the years 2006–2010 reveals major clones and an increase in serotype 1/2a. *BMC Infectious Diseases*.

[19] Gerner-Smidt P., Hise K., Kincaid J. (2006). PulseNet USA: a five-year update. *Foodborne Pathogens and Disease*.

[20] Salcedo C., Arreaza L., Alcalá B., de la Fuente L., Vázquez J. A. (2003). Development of a multilocus sequence typing method for analysis of *Listeria monocytogenes* clones. *Journal of Clinical Microbiology*.

[21] Zhang W., Jayarao B. M., Knabel S. J. (2004). Multi-virulence-locus sequence typing of *Listeria monocytogenes*. *Applied and Environmental Microbiology*.

[22] Chen Y., Zhang W., Knabel S. J. (2005). Multi-virulence-locus sequence typing clarifies epidemiology of recent listeriosis outbreaks in the United States. *Journal of Clinical Microbiology*.

[23] Chen Y., Zhang W., Knabel S. J. (2007). Multi-virulence-locus sequence typing identifies single nucleotide polymorphisms which differentiate epidemic clones and outbreak strains of *Listeria monocytogenes*. *Journal of Clinical Microbiology*.

[24] Knabel S. J., Reimer A., Verghese B. (2012). Sequence typing confirms that a predominant *Listeria monocytogenes* clone caused human listeriosis cases and outbreaks in Canada from 1988 to 2010. *Journal of Clinical Microbiology*.

[25] Lomonaco S., Verghese B., Gerner-Smidt P. (2013). Novel epidemic clones of Listeria monocytogenes, United States, 2011. *Emerging Infectious Diseases*.

[26] Tamura K., Stecher G., Peterson D., Filipski A., Kumar S. (2013). MEGA6: molecular evolutionary genetics analysis version 6.0. *Molecular Biology and Evolution*.

[27] Doumith M., Buchrieser C., Glaser P., Jacquet C., Martin P. (2004). Differentiation of the major *Listeria monocytogenes* serovars by multiplex PCR. *Journal of Clinical Microbiology*.

[28] MVLST database, https://sites.google.com/site/mvlstdatabase/

[29] ISTAT http://www.istat.it/it/istituto-nazionale-di-statistica.

[30] GenBank sequence database, http://www.ncbi.nlm.nih.gov/genbank/

[31] Garrido V., Torroba L., García-Jalón I., Vitas A. I. (2008). Surveillance of listeriosis in Navarre, Spain, 1995–2005—epidemiological patterns and characterization of clinical and food isolates. *Eurosurveillance*.

[32] MacDonald P. D. M., Whitwam R. E., Boggs J. D. (2005). Outbreak of listeriosis among Mexican immigrants as a result of consumption of illicitly produced Mexican-style cheese. *Clinical Infectious Diseases*.

[33] Wagner M., Allerberger F. (2003). Characterization of *Listeria monocytogenes* recovered from 41 cases of sporadic listeriosis in Austria by serotyping and pulsed-field gel electrophoresis. *FEMS Immunology and Medical Microbiology*.

